# The Roles of Attentional Shifts and Attentional Reengagement in Resolving The Spatial Compatibility Effect in Tactile Simon-like Tasks

**DOI:** 10.1038/s41598-018-27114-9

**Published:** 2018-06-08

**Authors:** Wanting Zheng, Lihan Chen

**Affiliations:** 1 School of Ophthalmology & Optometry, School of Biomedical Engineering, Wenzhou Medical University, Wenzhou, 325035 China; 2 School of Psychological and Cognitive Sciences and Beijing Key Laboratory of Behavior and Mental Health, Peking University, Beijing, 100871 China; 3 Key Laboratory of Machine Perception (Ministry of Education), Peking University, Beijing, China

## Abstract

The Simon effect refers to the acceleration of choice responses when the target position and response location are consistent compared with scenarios in which they are inconsistent, even if the target position is not relevant to the response. Here, we provide the first demonstration that the tactile Simon-like effect operates in an attention-shifting manner. In unimodal scenarios (Experiments 1–4), for the tactile direction task, the spatial compatibility effect was absent in the focused-attention condition but maintained in the divided-attention condition. For the tactile localization task, this pattern was reversed: the spatial compatibility effect occurred for the focused-attention condition but was reduced/absent in the divided-attention condition. In the audiotactile interaction scenario (Experiment 5), the reaction times (RTs) for discriminating the tactile motion direction were prolonged; however, a spatial compatibility effect was not observed. We propose that the temporal course of resolving conflicts between spatial codes during attentional shifts, including attentional reengagement, may account for the tactile Simon-like effect.

## Introduction

Tactile information processing via the hands is vital for daily life and environmental exploration. For example, dexterous grasping of an object requires accurate encoding of the spatial locations between fingers and the critical surface points of the object. During such grasping, encoding absolute (environment-based) and relative (anatomy-based) spatial locations contributes to successfully performing the appropriate action. In the perception-action loop, we perceive multiple properties of tactile events, such as intensity, texture and orientation, in addition to spatial location. However, certain task-irrelevant properties may facilitate or interfere with the discrimination of task-relevant properties and thus influence the performance of actions. These interactions among different properties are commonly observed via the Simon effect^1–3^.

The Simon effect refers to the facilitation of choice responses when the target position and response side are consistent compared with scenarios in which they are inconsistent, even if the target position is irrelevant to the response (for a review, see^3^). Numerous studies have addressed the phenomenological findings of different sensory modalities, as well as the potential encoding mechanisms and neuropsychological underpinnings of this effect^4–8^. One influential hypothesis, the attention-shifting hypothesis, states that the attentional landing of a certain spatial location (or a certain finger) imposes salience and processing priority; thus, the spatially congruent task (property) is in the focus of attention and receives facilitated processing. In contrast, the spatially incongruent target stimulus is outside the focus of attention, and processing of the stimulus’ properties (such as intensity) is hampered, thus producing the Simon effect^9,10^. Following this logic, a shift in attentional focus would re-assemble and re-organize attention-emergent processing and cause the otherwise absent Simon effect to occur again. Indeed, evidence from the visual domain has confirmed the role of attentional shifting in the Simon effect. On the other hand, in the same theoretical framework, the attentional selection account places an emphasis on the time course of attentional selection. According to the early attentional selection hypothesis, the programming of an attentional shift toward the stimulus location rather than the stimulus position itself (as in coding hypothesis^11,12^ causes the stimulus-response (S-R) compatibility pattern to emerge in the Simon task^13,14^. According to the late attentional selection hypothesis, selecting a location in space primes action toward that location and eventually shifts attention in that direction^15^.

Another influential theoretical framework, the referential coding theory, emphasizes a perception-for-action system and states that the Simon effect results from the coding of all S-R features (with the current focus of attention) and ensuing response selection. This framework explicitly accounts for differential spatial references across different sensory modalities. For example, the tactile modality exhibits specific spatial reference frameworks, both external (environment-oriented) and internal (self-oriented), and these frameworks are different from those of the visual (retinotopic) and auditory (head-centered) modalities. In contrast to vision, which is a distal modality, tactile sensations originate from stimuli that directly impinge on the bodily surface. The processing of tactile events intrinsically gives stronger weight to an anatomical frame of reference (‘body code’)^16–18^, and the effect of this body code is difficult to cancel out when multiple potential references co-exist and even conflict. Indeed, some authors have shown that tactile Simon effects adhere to an anatomical frame of reference^18–20^, not to external ones such as vision^12,21^ and audition. Wallace first postulated that the body (e.g., the responding hand) represents a source of spatial information that should be treated as a stimulus itself. According to the coding hypothesis by Wallace (1971), the hand is represented in the form of a ‘body code’ and can be associated with a location in space with the references updated as the hand (fingers) move from one position to another^12^. This referential coding hypothesis offers an effective framework to explain the S-R compatibility pattern from spatial updating or modality switches^18^.

However, most of the above studies used hands as response effectors, which complicates the direct examination of the mechanism of ‘body codes’, wherein the hands (fingers) are indeed the recipients of tactile stimuli. On the other hand, studies of S-R compatibility and the Simon effect have thus far employed static stimuli almost exclusively. In this case, the spatial correspondence (whether relevant or not in a task) is unambiguous. For unisensory (tactile) moving stimuli, however, correspondence may be associated either with the position from which the motion starts (position compatibility) or with the direction in which the stimulus moves (direction compatibility)^5^. Moreover, in a cross-modal scenario, visual and auditory events trigger a spatial code based on external coordinates and tactile events trigger spatial representations based on anatomical coordinates. These two coordinates are incongruent, but no empirical studies have investigated how temporal asynchrony between auditory and tactile events would modulate the spatial compatibility effect of tactile perception on the hands/fingers.

Therefore, to address the above empirical research questions, in the present study we implemented five experiments to show how the spatial compatibility of stimuli within single modality (tactile modality) and across modalities (tactile vs. auditory modality) affect the discrimination for target tactile events. Specifically, in the unimodal scenario, we implemented four experiments and manipulated the attention-shift and predictability of the tactile events. In Experiment 1, participants focused their attention on the two consecutive taps on one hand (the first tap appeared on either the left middle or right middle finger, and the second one appearing on the finger next to the above - forefinger or ring finger), hereafter we named it as ‘FA fixed’ condition.In Experiment 2, participants divided their attention on the three taps, with the first two taps appearing simultaneously on the two middle fingers and the third one appearing on the forefinger or the ringfinger on either hand, hence we named the condition as ‘DA fixed’. Experiment 3 was similar to Experiment 1 except that the two taps appeared randomly on the fingers (expect the thumb) within one hand (‘FA random’). Experiment 4 was similar to Experiment 2 except that the first two simultaneous taps were not always on both middle fingers but were from one of the following four combinations (left middle finger/right middle finger; left middle finger/right forefinger; left forefinger/right middle finger; left forefinger/right forefinger), the third tap appeared randomly on the finger next to the first tap appearing previously. We named the condition in Experiment 4 as ‘DA random’. Therefore, with the above settings, we were able to pit the spatial compatibility in *motion direction* against *localization*, in terms of the spatial congruency with respective to the hand. For example, the left tactile motion stream in left hand, is spatially congruent for motion direction, but is incongruent in terms of the starting position (right).

In crossmodal scenario (experiment 5), we examined how potential conflicts are resolved when observers discriminated the direction of tactile motion in the presence of auditory distractors with a temporal delay between auditory and tactile events (Experiment 5). In both unisensory and crossmodal scenarios, we examined the roles of attention shifting and referential coding in resolving the conflicts/uncertainties of spatial references.

In all the above settings, participants responded with foot pedals. Therefore, the response effectors were dissociated from their hands. In addition to spatial coding, the potential time course over which conflicting spatial codes attention and attentional shift/selection were resolved could determine the magnitude of spatial compatibility effect. We aimed to reconcile attention-shifting account and referential coding account in terms of the constraints of the time course for attentional selection.

The attention-shifting account (after^13,14^) is basically an early-selection approach, whereas the referential coding account (after^2^) implies a late-selection approach to attention. The localization of tactile events in different fingers was more demanding than the discrimination of motion direction^22–26^. According to the referential coding account, the time-demand (with longer reaction time) of the *localization* task and the relatively longer interval between cross-modal cues and the target tactile events would trigger a spatial compatibility effect. In contrast, for motion direction discrimination without cross-modal cues, an early and prompt attentional modulation would make an attention-shifting account viable, and we would expect a spatial compatibility effect.

## Results

Because the individual participants’ accuracy was above 95%, we focused on analyzing the reaction time for the five experiments.

### Unimodal Tasks: compatibility effect

To give a concise picture of the spatial compatibility effect, we further collapsed and sorted all the data into compatible and incompatible patterns. In direction task, the compatible pattern refers to that the motion direction (leftward vs. rightward) is congruent with the hand location (left vs. right hand). In the location task, the compatible pattern refers to that the relatively starting location of the tap (left vs. right) is congruent with the hand location

**Figure 1:**
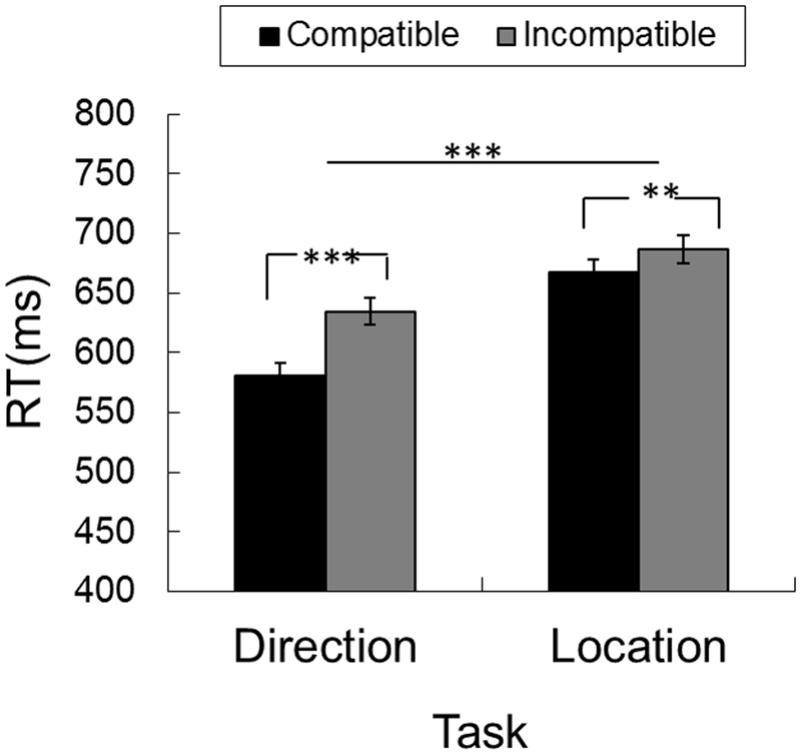
The compatibility effect in both ‘direction’ and ‘location’ tasks. The error bars represent standard errors. ^**^p < 0.01; ^***^p < 0.0001.

The RTs for compatible and incompatible conditions were 624.0 ± 7.7 ms and 660.6 ± 8.2 ms, the main effect of compatibility was significant (*F*(1,143) = 62.983, *p* = 5.520 × 10^−13^, ${\eta }_{g}^{2}=0.306$). The RT for discriminating motion direction (607.8 ± 10.7 ms) was faster than the one in localization (676.8 ± 10.8 ms) [(*F*(1,143) = 20.526, *p* = 1.232 × 10^−5^, ${\eta }_{g}^{2}=0.126$)]. The interaction effect between the factors of task and compatibility was significant (*F*(1,143) = 13.833, *p* = 2.862 × 10^−4^, ${\eta }_{g}^{2}=0.088$). Simple effect analysis indicated that the compatibility effect was observed in both ‘direction’ (*p* = 7.316 × 10^−14^) and ‘location’ (*p* = 0.004) tasks (Fig. 1). The three-way interaction between factors of task, compatibility and experiments was significant (*F*(3,143) = 47.442, *p* = 2.418 × 10^−21^, ${\eta }_{g}^{2}=0.499$) (Table 1).

**Table 1:** The reaction times (RTs), with associated standard errors, as a function of tasks, conditions and compatibilities.

Task	Condition	Compatible	Incompatible
Direction	FA-fixed	564.2(21.0)	550.3(22.5)
	DA-fixed	557.3(21.0)	668.4(22.5)
	FA-random	578.1(22.2)	574.0(23.7)
	DA-random	624.2(22.2)	746.1(23.7)
Location	FA-fixed	612.0(22.8)	679.2(24.4)
	DA-fixed	662.3(23.5)	656.2(25.1)
	FA-random	638.7(20.5)	700.7(21.9)
	DA-random	755.3(20.5)	709.9(21.9)

### Detailed analysis across all experimental conditions

We conducted ANOVA analysis with the factors of space (left vs right hand), motion direction and task (direction or location judgment).The mean reaction times (RTs) for leftward were longer (648.9 ± 7.9 ms) than those (635.7 ± 7.9 ms) for rightward motion (*F*(1,143) = 10.305, *p* = 0.002, ${\eta }_{g}^{2}=0.067$). The mean RTs for stimuli occurring at the left hand (left-hand space) and at the right hand (right-hand space) were 641.9 ± 8.2 ms and 642.6 ± 7.5 ms, respectively (*F*(1,143) = 0.027, *p* = 0.870, ${\eta }_{g}^{2}=0.000$). Generally, the RT for motion direction judgments (607.8 ± 10.7 ms) was faster than the discrimination of stimulus location (676.8 ± 10.8 ms) (*F*(1,143) = 20.526, *p* = 1.231 × 10^−5^, ${\eta }_{g}^{2}=0.126$). The three-way interaction among space, motion direction and task (direction or location judgment) was significant (*F*(1,143) = 62.983, *p* < 0.001^−13^, ${\eta }_{g}^{2}=0.306$). An analysis of the simple effects pertaining to the interaction among space, direction and task (motion discrimination vs. localization) showed that for stimuli occurring in the left-hand space with leftward motion, the RTs for motion judgment (591.2 ± 11.4 ms) were faster than those for localization (688.7 ± 11.6 ms) (*p* = 1.599 × 10^−8^). In addition, for stimuli occurring in the right-hand space with rightward motion, the RT for motion judgment (570.6 ± 10.8 ms) was faster than that for localization (684.3 ± 10.9 ms) (*p* = 9.546 × 10^−12^). This pattern further indicated that motion direction discrimination was faster than motion stream starting-point localization, independent of the spatial compatibility effect.

The main effect of the experimental manipulations was significant (*F*(1,143) = 9.584, *p* = 8.301 × 10^−6^, *η*^2^_g_ = 0.167,). The mean RTs for Experiment 1 (FA fixed), Experiment 2 (DA fixed), Experiment 3 (FA random) and Experiment 4 (DA random) were 601.4 ± 15.4 ms, 636.0 ± 15.6 ms, 622.9 ± 15.0 ms and 708.9 ± 15.0 ms, respectively. Bonferroni-corrected comparisons showed that the RTs for the FA fixed, DA fixed and FA random conditions were significantly faster than those of the DA random condition (*p* < 0.01 for all conditions). However, the interaction between factors of experimental conditions and tasks (motion discrimination vs. localization) was not significant (*F*(3,143) = 0.708, *p* = 0.549, ${\eta }_{g}^{2}=0.015$.

### Motion Direction Task

We conducted ANOVA analysis with the factors of space (left vs right hand), motion direction (leftward vs. rightward). The RTs for discriminating the direction of the motion stream in the left- and right-hand spaces were 608.7 ± 11.5 ms and 606.9 ± 10.4 ms, respectively (*F*(1,72) = 0.111, *p* = 0.740, ${\eta }_{g}^{2}=0.002$). The RTs for judging leftward motion (617.2 ± 11.1 ms) were longer than those for rightward motion (598.4 ± 11.2 ms) (*F*(1,72) = 8.224, *p* = 0.005, ${\eta }_{g}^{2}=0.103$). Overall, observers had an initial bias for rightward motion, which may be due to their preference for motion direction such as in reading habits (from left to right).

**Figure 2:**
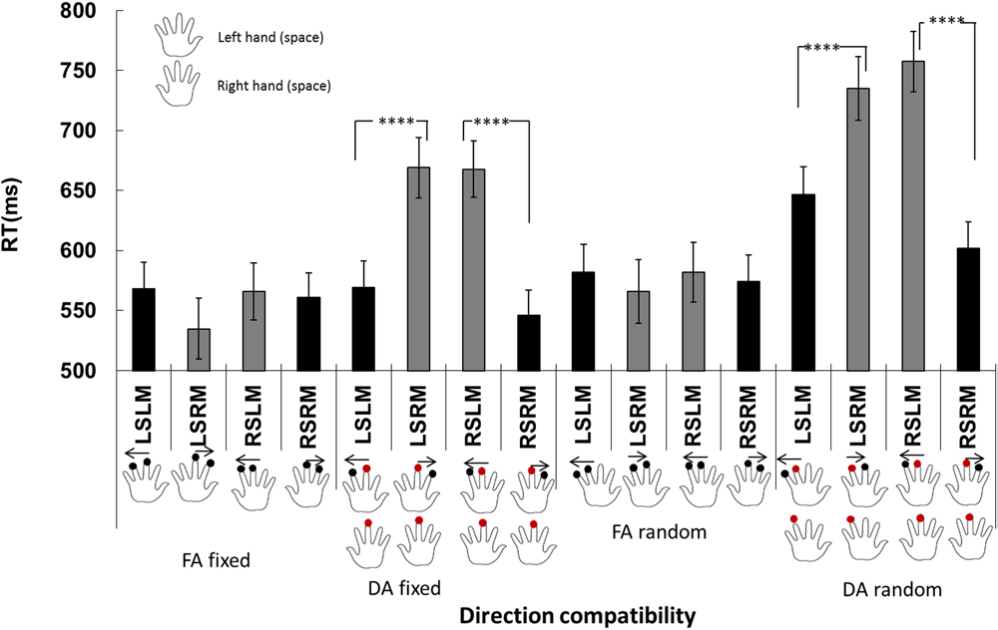
Reaction times for the motion direction-based Simon-like tactile spatial compatibility effect. LSLM: tactile stimuli occurred on the left and the direction of motion was leftwards; LSRM: left side, rightward motion; RSLM: right side, leftward motion; RSRM: right side, rightward motion. **FA fixed**: tactile taps with focused attention (Exp. 1); the direction of tactile motion was taps from the middle finger to forefinger. **DA fixed:** tactile taps with divided attention; the direction of tactile motion was from middle finger to forefinger (the motion stream appeared either at the left hand or at the right hand), but the first two simultaneous taps occurred at both middle fingers (Exp. 2). **FA random**: taps were distributed randomly (except the thumb) on the fingers in either hand (Exp. 3). **DA random**: tactile taps with divided attention and random taps (Exp. 4). Two taps in a motion stream were distributed randomly (except the thumb) on the fingers in either hand; however, the first two simultaneous taps appeared on the left middle finger/right forefinger or left forefinger/right middle finger. The arrow above the hand shows the direction of tactile apparent motion. The red dots on both hands indicate the simultaneous two taps. The black columns indicate ‘congruent’ conditions, while the gray columns indicated the ‘incongruent’ conditions. Error bars indicate one standard error of the mean. ^***^p < 0.001; ^****^p < 0.0001.

The RTs for discriminating leftward and rightward motion in the left-hand space were 591.3 ± 11.1 ms and 626.2 ± 13.8 ms, respectively, and the RTs for discriminating leftward motion and rightward motion in the right-hand space were 643.2 ± 12.6 ms and 570.6 ± 10.7 ms, respectively. The interaction between space and motion was significant (*F*(1,72) = 50.337, *p* = 7.387 × 10^−10^, ${\eta }_{g}^{2}=0.011$). A further simple effect analysis showed that in the left-hand space, the RTs for leftward motion discrimination were shorter than those for rightward motion (*p* = 5.415 × 10^−4^), whereas in the right-hand space, the pattern was reversed, with RTs for rightward motion being shorter than those for leftward motion (*p* = 1.196 × 10^−9^) (Fig. 2). Therefore, spatial (hand) and directional congruency contributed to the observed spatial compatibility effect.

The main effect of Experiments (1–4) was significant (*F*(3,72) = 6.871, *p* = 3.905 × 10^−4^, ${\eta }_{g}^{2}=0.223$). The mean RTs for Exps. 1–4 were 557.3 ± 20.7 ms, 612.8 ± 20.7 ms, 576.0 ± 21.9 ms, and 685.2 ± 21.9 ms. Bonferroni-corrected comparison showed that significant differences between the cohorts were observed as follows: RT_exp 1_ < RT_exp 4_ (*p* = 3.871 × 10^−4^) and RT_exp 3_ < RT_exp 4_ (*p* = 0.004). The interaction among space (hand), motion direction and experiment was significant (*F*(3,72) = 23.066, *p* = 1.433 × 10^−10^, ${\eta }_{g}^{2}=0.490$). A further analysis of simple effects showed that the typical spatial compatibility effect was only observed in Experiments 2 and 4, in which participants had their attention divided by task-irrelevant tactile taps before performing the relevant task of discriminating tactile apparent motion. Specifically, in the left-hand space for Exp. 2, the RTs for leftward and rightward motion were 568.9 ± 21.7 and 669.0 ± 26.8 ms, respectively (*p* = 1.047 × 10^−6^), whereas in the right-hand space, the RTs for leftward motion and rightward motion were 667.7 ± 24.4 ms and 545.8 ± 20.8 ms, respectively (*p* = 6.538 × 10^−8^). For Experiment 4, in the left-hand space, the RTs for leftward and rightward motion were 646.6 ± 22.9 ms and 734.8 ± 28.2 ms (*p* = 2.919 × 10^−5^), whereas in the right-hand space, the RTs for leftward and rightward motion were 757.4 ± 25.8 ms and 601.9 ± 21.9 ms (*p* = 3.202 × 10^−10^). These results suggested that in addition to the spatial factors (hand and motion direction) modulating the spatial compatibility effect, divided attention by task-irrelevant tactile events generally prolonged RTs and magnified the spatial compatibility effect. The potential contributor is the time course of attentional focus and attentional (re) engagement. The potential underlying mechanisms are addressed further in the Discussion.

### Localization Task

**Figure 3:**
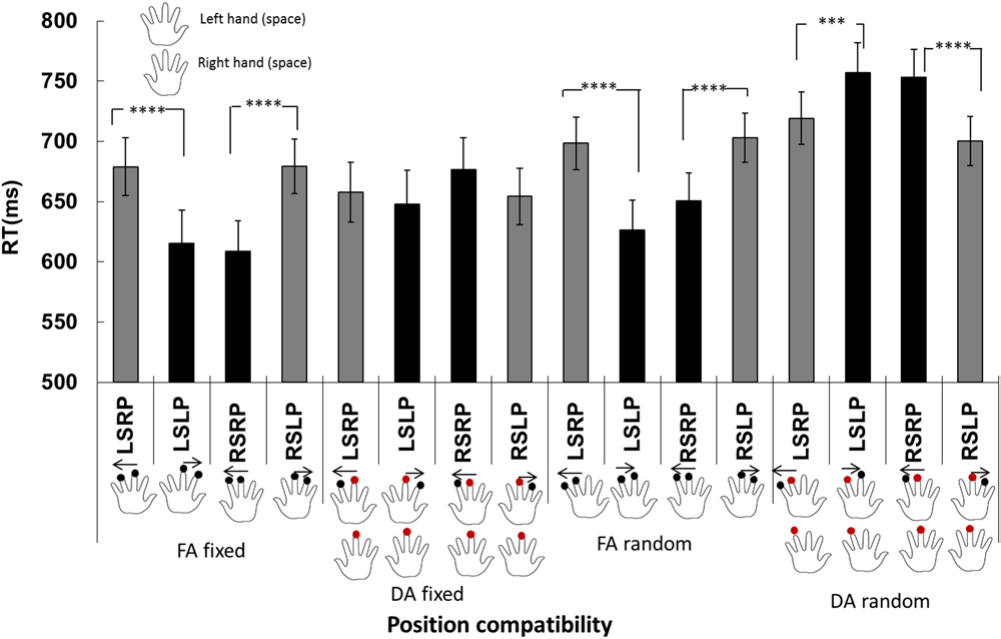
Reaction times for the position-based Simon-like tactile spatial compatibility effect. **LSRP**: tactile stimuli occurred on the left hand, and the starting position of the motion stream was to the right; **LSLP**: tactile stimuli occurred on the left hand, and the starting position of the motion stream was to the left; **RSRP**: tactile stimuli occurred on the right hand, and the starting position of the motion stream was to the right. **RSLP:** tactile stimuli occurred on the right hand, and the starting position of the motion stream was to the left. The meanings of the four conditions (Exps) of FA fixed, DA fixed, FA random and DA random were the same as in Fig. 1. The arrow above the hand shows the direction of the tactile apparent motion. The red dots on both hands indicate the simultaneous two taps. The black columns indicate ‘congruent’ conditions while the gray columns indicate the ‘incongruent’ conditions. Error bars indicated one standard error of the mean. ^***^p < 0.001; ^****^p < 0.0001.

We conducted ANOVA analysis with the factors of space (left vs right hand) and relatively starting position (left vs. right). The RTs for discriminating the starting tap of the motion stream in the left-hand and right-hand spaces were 675.3 ± 11.5 ms and 678.3 ± 10.8 ms, respectively (*F*(1,71) = 0.348, *p* = 0.557, ${\eta }_{g}^{2}=0.005$). The RTs for judging right and left starting positions were 680.5 ± 11.2 ms and 673.1 ± 11.1 ms, respectively (*F*(1,71) = 2.362, *p* = 0.129, ${\eta }_{g}^{2}=0.032$). The two-way interaction between starting position and space (hand) was significant (*F*(1,71) = 13.979, *p* = 3.711 × 10^−4^, ${\eta }_{g}^{2}=0.165$). In the left-hand space, the RTs for right and left starting positions were 688.7 ± 11.8 and 661.8 ± 12.2 ms, respectively, whereas in the right-hand space, the RTs for the right and left starting positions were 672.3 ± 11.8 ms and 684.3 ± 10.9 ms, respectively. Further simple effect analysis showed that for the left hand (space), the RTs in the left starting position were shorter than those in the right starting position (*p* = 2.873 × 10^−4^) (Fig. 3).

However, no difference was observed between the RTs for the two starting positions when the stimuli were presented in the right-hand space (*p* = 0.099). Conversely, for the right starting position, the RTs for stimuli occurring in the left-hand space were longer than those for stimuli occurring in the right-hand space (*p* = 0.037). In contrast, for the left starting position, the RTs for stimuli occurring in the left-hand space were faster than those for stimuli occurring in the right-hand space (*p* = 0.002). These results suggested that congruency between (updated) relative location with respect to the midline of the hand and the absolute location (left vs. right) of the hand led to the tactile spatial compatibility effect.

The main effect of Experiments (1–4) was significant (*F*(3,71) = 3.333, *p* = 0.024, ${\eta }_{g}^{2}=0.123$). The mean RTs for Exps. 1–4 were 645.6 ± 22.7 ms, 659.2 ± 23.4 ms, 669.7 ± 20.4 ms, and 732.6 ± 20.4 ms. Bonferroni-corrected comparison showed that significant differences between the cohorts were observed as follows: RT_exp 1_ < RT_exp 4_ (*p* = 0.034). The interaction among space (hand), motion direction and experiment was significant (*F*(3,71) = 29.336, *p* = 1.895 × 10^−12^), ${\eta }_{g}^{2}=0.553$. A subsequent analysis of simple effects indicated that the typical spatial compatibility effect was observed in Experiments 1, 3 and 4. Specifically, in the left-hand space for Exp. 1, the RTs for leftward and rightward tactile motion (hence, right and left starting location, respectively) were 679.1 ± 24.7 ms and 615.5 ± 25.5 ms (*p* = 4.794 × 10^−5^) whereas in the right-hand space, the RTs for leftward and rightward motion (hence, right and left starting location, respectively) were 608.6 ± 24.6 ms and 679.4 ± 22.9 ms, respectively (*p* = 1.089 × 10^−5^). For Experiment 3, in the left-hand space, the RTs for leftward and rightward motion were 698.5 ± 22.2 ms and 626.7 ± 22.9 ms (*p* = 7.400 × 10^−7^), whereas in the right-hand space, the RTs for leftward and rightward motion were 650.7 ± 22.2 ms and 702.9 ± 20.6 ms, respectively (*p* = 2.241 × 10^−4^). For Experiment 4, in the left-hand space, the RTs for leftward and rightward motion were 719.3 ± 22.2 ms and 757.2 ± 22.9 ms, respectively (*p* = 0.005), whereas in the right-hand space, the RTs for leftward and rightward motion were 753.5 ± 22.2 ms and 700.5 ± 20.6 ms, respectively (*p* = 1.873 × 10^−4^). For Experiment 2, in the left-hand space, the RTs for leftward and rightward motion were 658.0 ± 25.4 ms and 648.0 ± 26.2 ms, respectively (*p* = 0.510), whereas in the right-hand space, the RTs for leftward and rightward motion were 676.6 ± 25.4 ms and 654.4 ± 23.6 ms, respectively (*p* = 0.155).

For the localization task, spatial compatibility was absent in the DA fixed condition (Exp. 2) and was reversed in the DA random condition (Exp. 4), i.e., RTs in the ‘incongruent’ condition were shorter than those in the ‘congruent’ condition. We reconcile the seemingly contradictory findings in terms of uncertainty of the tactile events and the cost of resolving the spatial representations of tactile events. For the DA fixed condition, when the first two simultaneous taps were given and the tactile events had been determined, there remained two potential directions for tactile motion on one hand; hence, the perception of the succeeding tactile events (locations) became more certain. Therefore, the conflicts of spatial coordinates were reduced, and the spatial compatibility effect was weakened.

For the DA random condition, however, the first two simultaneous but randomized taps indeed increased the uncertainty for locations of tactile events and led to conflicts of the congruency between hand (left vs. right) and the relative starting tactile location. Therefore, the RTs in DA random (Exp. 4) were generally longer than those in the other three conditions. Note that the first two simultaneous taps appeared on the left middle finger/right forefinger or left forefinger/right middle finger; for the congruent conditions of ‘LSLP’ and ‘RSRP’ (Fig. 3), the discrimination of tactile location might be subject to the inhibition of return, or the slowing of responses to stimuli presented at the same location as a preceding cue^27–29^.

### Cross-modal task

**Figure 4:**
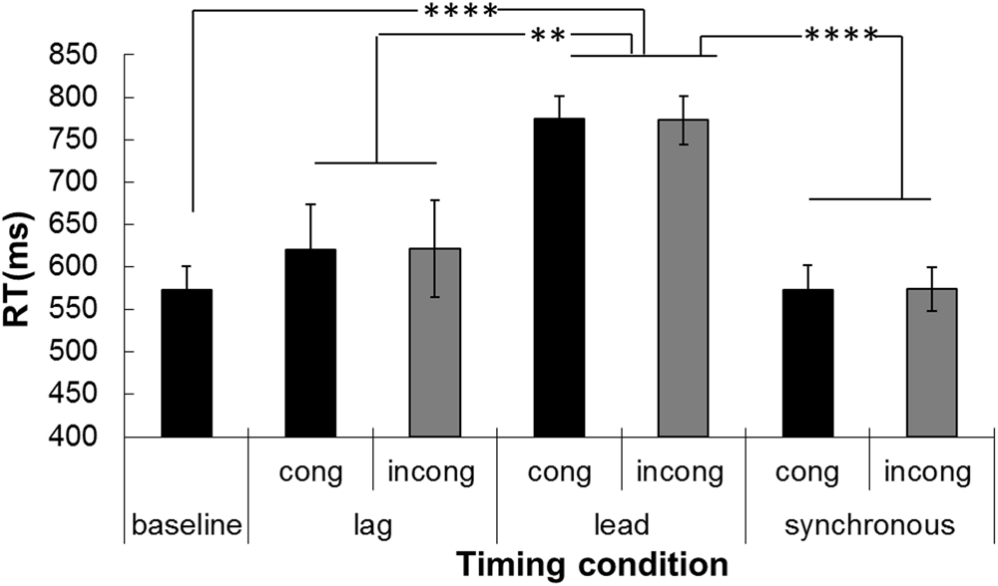
RTs for discriminating the direction of the tactile motion stream under audiotactile congruent or incongruent motion stream conditions and under baseline, auditory pre-cue (‘lead’), synchronous auditory cue (‘synchronous’) and post-cue (‘lag’) conditions. The red bars indicate the statistically significant results between ‘baseline’ and ‘lead’ conditions.

The mean RTs for the baseline, lag, lead and synchronous conditions were 572.7 ± 28.3 ms, 620.8 ± 55.4 ms, 774.1 ± 27.1 ms and 573.2 ± 27.0 ms, respectively (F(3,33) = 29.605, *p* = 1.771 × 10^−9^, ${\eta }_{g}^{2}=0.729$). Bonferroni-corrected comparisons indicated that the RTs in the baseline condition were significantly shorter than those in the lead condition (*p* = 3.415 × 10^−8^). The RTs in the lead condition were significantly longer than those in the lag (*p* = 0.009) and synchronous conditions (*p* = 4.465 × 10^−8^) (Fig. 4).

For the sound-only conditions, the main effect of stimulus type was significant (*F*(2,22) = 25.801, *p* = 1.702 × 10^−6^, ${\eta }_{g}^{2}=0.701$). However, the main effect of congruency was not significant (*F*(1,11) = 0.003, *p* = 0.958, ${\eta }_{g}^{2}=0$). The RT for the congruent condition was 655.8 ± 35.2 ms, and that for the incongruent condition was 656.3 ± 35.5 ms. The interaction between stimulus type and congruency was also not significant (*F*(2,22) = 0.073, *p* = 0.930 ${\eta }_{g}^{2}=0.007$.

Therefore, in contrast to previous studies on the cross-modal spatial compatibility effect, we did not find a congruency effect of the spatial correspondence between auditory events and tactile events. These null findings were probably due to the different spatial reference frameworks adopted in the current experimental setting, which did not lead to observable conflicts in spatial coordinates. The auditory events given to both ears were referred to head-centered spatial coordinates and are more body-center oriented^30–32^, while the two tactile events appearing on a single hand mobilize an anatomical frame of reference^18–20^, and the two taps could refer to each other with less emergent body-center oriented^33,34^. However, the temporal asynchrony of pre-cued auditory events led to a general interference effect of perceiving events from a third modality, such as in tactile perception^35–37^.

## Discussion

Ehrenstein (1994) examined the S-R compatibility and Simon effects for real visual motion. They found strong compatibility effects for both position and direction tasks. A Simon effect, however, occurred only when position was task-irrelevant (namely, divided attention upon the first two simultaneous lights to the left and right of the central fixation), and no Simon effect was observed in the focused-attention condition (i.e., motion onset coincided with stimulus onset). Here, we observed a similar spatial compatibility effect in the tactile modality. The compatibility effect is dependent on task demands and attentional processing. The compatibility effect was surprisingly absent when participants discriminated the direction of tactile motion across hand spaces, with either congruent or incongruent hands (left vs. right) and the direction of tactile motion (leftward vs. rightward). In contrast, for the localization task, we found a robust spatial compatibility effect when participants focused their attention on the starting location of the tactile taps (Experiments 1 and 3) without interference from the task-irrelevant taps, as in Experiments 2 and 4.

Generally, tactile motion direction was discriminated faster relative to the participants’ reporting of the starting position of the tactile taps. For moving stimuli, the correspondence between stimulus and response may be associated either with the position from which the motion starts (position compatibility) or with the direction in which the stimulus moves (direction compatibility). The finding that the RTs for motion direction discrimination were faster than those for localization is consistent with previous evidence. Michaels (1988) asked participants to use a joystick movement to respond to visual apparent motion. Participants had to respond either to the starting position of apparent motion or to its destination. When subjects responded to the starting position, a standard spatial compatibility effect was observed, regardless of the destination; however, when they responded to the destination, the responses were faster at the destination location, even when the spatial location was incompatible (destination compatibility)^38^.

The Simon-like effect is currently explained in terms of the generation and use of spatial codes, with two influential theoretical frameworks: the attentional-shift hypothesis and the referential coding account. The attentional-shift hypothesis states that a spatial code is generated when a shift in spatial attention occurs toward the location occupied by the imperative stimulus^9,10,39^. The referential coding account holds that a spatial code is formed by relating the imperative stimulus to a reference frame^2,3^. The attentional orienting account fulfills a crucial role for the spatial compatibility effect and indicates that in the absence of attentional orienting, response priming should not occur, and thus, a spatial compatibility effect should not be observed^40^. In other words, if attention is already directed toward the relevant location, then the cue induces no response benefit. However, in the case of divided attention, attention provides either a benefit or a cost by activating the corresponding or the non-corresponding action/task.

The present findings may be explained by reconciling the attention-shifting account and the referential coding account in terms of the constraints of the time course during attentional selection. The attention-shifting account is basically an early-selection approach, whereas the referential coding account implies a late-selection approach to attention. In the current tasks, the discrimination of tactile motion was faster than the identification of the location of the starting position of the tactile motion. Therefore, for motion direction discrimination, fast and prompt attentional modulation would make the attention-shifting account largely subservient to the spatial compatibility effect. However, for the localization task, the late selection of the targets engendered the spatial compatibility effect, and even reversed the ‘congruency’ effect (for DA random, ‘localization’ task). This time course includes resolving spatial compatibility and dissipating interfering spatial codes^5,41,42^. If attention is and remains focused on a location, then there should be no ‘response’ priming, and no spatial compatibility effect should be observed. Moreover, attention must be disengaged from the nonmoving location at motion onset and then switched to the moving side, which requires the additional step of disengaging attention^43,44^. In the current study, the discrimination of tactile direction in the focused-attention condition entails no need to (re)orient attention because the onset of the first tap was followed immediately by a second tap on the same hand to render the apparent motion perception. Therefore, additional conflict was not introduced between the initial attention landing of the first tap and the subsequent focus on the second tap. In the divided-attention condition, participants had to disengage their attention from the first tap (or from the two simultaneous taps) and reorient to the target motion stream. The attention shift thus imposes a cost and leads to the spatial compatibility effect. Generally, as we have demonstrated, the RTs for discriminating tactile motion direction are shorter than those for reporting the starting location of the tactile motion stream; thus, participants tended to respond promptly, even before attentional disengagement had completed. Therefore, conflict between spatial locations still occurs because of the incongruent spatial representations of taps associated with the task-irrelevant and target hands, which contributes to the observed spatial compatibility effect.

However, the results for the tactile localization task provided a different picture. For the focused-attention protocol, four potential scenarios (left hand/left position, left hand/right position, right hand/left position and right hand/right position) were introduced for discriminating the starting position of the taps as left or right. The uncertainty in the focused-attention condition was higher than that in the divided-attention condition because after approximately 300 ms, attentional orienting had already completed, and the participants’ attention was then re-engaged to the target task (which explains why the RTs in the localization task were longer than those in the direction task). Moreover, in the divided-attention task, two options (left vs. right) were provided for discriminating the starting position, and the hand for the target task was fixed after attention was re-engaged. Thus, the likelihood of spatial (representation) conflicts was low, and we observed a smaller spatial compatibility effect in the localization task. However, the surprising reversal of the ‘congruency’ effect in the DA random condition in the localization task suggested that another mechanism might be taking effect. Note that the first two simultaneous taps appeared on the left middle finger/right forefinger or the left forefinger/right middle finger for the congruent conditions of ‘LSLP’ and ‘RSRP’ (Fig. 2). During the relatively longer time window (above 300 ms) after the onset of the tactile events, the discrimination of end tactile location is probably subject to the inhibition of return [33] or the slowing of responses to stimuli presented at the exactly the same location as a preceding cue. Hence, the so-called ‘congruent’ condition brought forth a cost of reaction time, and we indeed found a ‘reversal’ of RTs between ‘congruent’ and ‘incongruent’ mapping of hand (left vs. right) and starting relative location (left vs. right) of the first moving tap.

In cross-modal scenarios, previous studies have suggested that a supramodal spatial map is shared between various input and output modalities. The pre-cueing of sound signals produces strong interference with the processing of tactile apparent motion direction because cross-modal pre-cueing may cause slower attention shifts^41^. If attention is slowed, then the attention shift may not be completed, even at long stimulus onset asynchronies (SOAs), which may lead to cross-modal interference, as well as the spatial compatibility effect, as observed for tactile modality. However, in Experiment 5, we did not observe a spatial compatibility effect but rather found a general interference effect (prolonged RT). This intriguing result is likely due to distinct spatial reference frames between auditory and tactile events in the current setting. In Experiment 5, auditory stimuli were provided through earphones, whereas tactile stimuli were provided upon the hands. The head-centered spatial representation for sounds and the hand-centered spatial reference for tactile events are largely inconsistent. The participants were subject to greater temporal interference than spatial conflicts; therefore, performance was generally only reduced for discriminating the direction of tactile motion in the presence of a preceding auditory beep (with extended RT).

Recent studies on tactile spatial processing have also demonstrated the role of cognitive control^45^. Compared with visual stimulation, control over the spatial processing of tactile stimulation requires more resources or a different strategy to inhibit spatial processing when the stimulus is irrelevant. An increase in the interference component would enlarge the congruency effect because of a decrease in proactive control. We believe that participants may adopt different cognitive control strategies in the current tasks, although further empirical evidence is required to address this potentially influential factor.

In summary, we employed a tactile Simon-like spatial compatibility paradigm and found a double dissociation in the spatial compatibility effect between tactile motion direction and localization tasks. This double association is accounted for by the temporal course of resolving conflicting spatial codes during attentional shifts, including attentional reengagement, in addition to the alignment of the conflicting and interfering spatial coordinates.

## Methods

### Participants, Apparatus and Stimuli

A total of ninety-four participants (n = 20, 20, 21, 21, and 12 in Experiments 1–5) whose ages ranged from 18–33 years took part in the experiments. Since the number of experimental conditions was reduced in Experiment 5, we collected a smaller dataset than in the other four experiments. Nevertheless, we still obtained robust statistical power.

Informed consent was obtained from each participant. The study was approved by the Academic Affairs Committee of the School of Psychological and Cognitive Sciences at Peking University. All experiments were performed in accordance with the approved guidelines and regulations.

**Figure 5:**
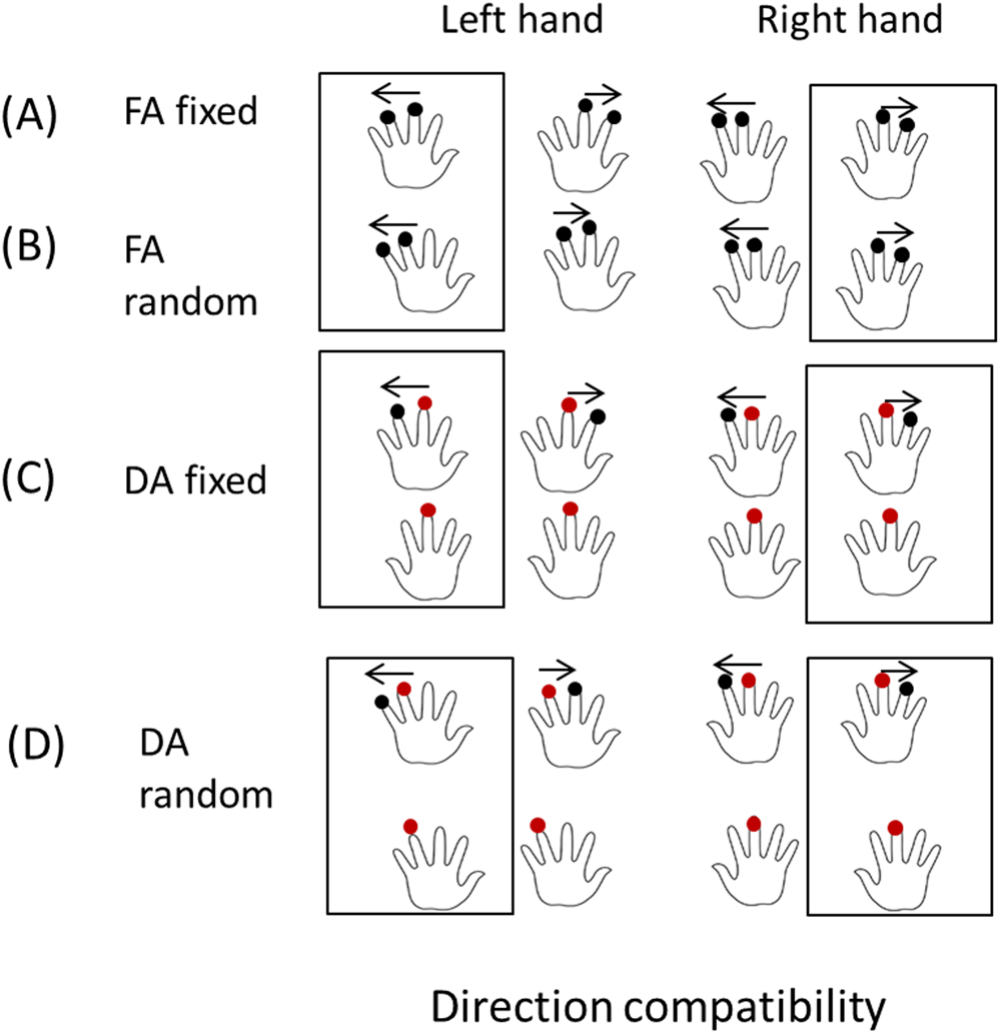
Schematic illustration of the Simon-like tactile directional compatibility effect. Oval dots indicate potential locations where tactile taps were generated. For each trial, only two taps occurred on one hand, except for the divided-attention condition (**C**,**D**), in which the first two taps (labeled as red dots) occurred at the same time across two hands. The task was to report the direction (leftwards or rightwards) of the tactile apparent motion. The four sub-figures illustrate the four sub-conditions. **FA fixed**: tactile taps with focused attention (Exp. 1); the direction of tactile motion was from the middle finger to the forefinger. **DA fixed:** tactile taps with divided attention; the direction of tactile motion was from the middle finger to the forefinger (the motion stream appeared either at the left hand or at the right hand), but the first two simultaneous taps occurred at both middle fingers (Exp. 2). **FA random**: taps were distributed randomly (except the thumb) on the fingers in either hand (Exp. 3). **DA random:** tactile taps with divided attention and random taps (Exp. 4). Two taps in a motion stream were distributed randomly (except the thumb) on the fingers in either hand; however, the first two simultaneous taps appeared on the left middle finger/right forefinger or the left forefinger/right middle finger. Tactile events within the rectangle indicate the congruent mapping of motion direction and space (of hand).

**Figure 6:**
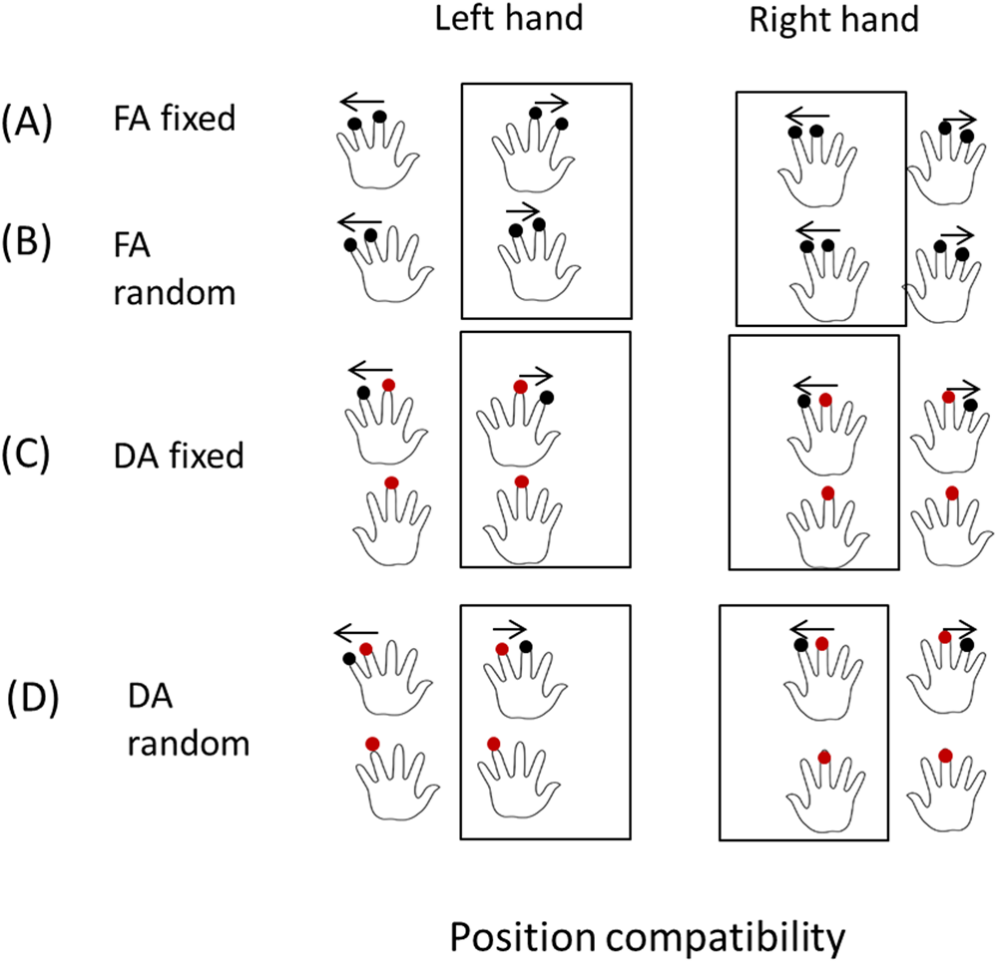
Schematic illustration of the Simon-like tactile position compatibility effect. Oval dots indicate potential locations where tactile taps were generated. For each trial, only two taps occurred on one hand, except for the divided-attention condition (**C,D**), in which the first two taps (labeled as red dots) occurred at the same time across two hands. The task was to report the starting location (left vs. right) of the tactile apparent motion. The four sub-figures illustrate the four sub-conditions: **FA fixed**, **DA fixed**, **FA random** and **DA random** (with the same settings as in Fig. 3). The tactile events within the rectangle indicate the congruent mapping of the starting location of the tactile motion stream and space (of hand).

Eight solenoid tactile actuators (Heijo Box, Heijo Research Electronics, UK) were used to present the tactile stimuli. The maximum contact area for each tap was approximately 4 mm^2^, and the maximum output was 3.06 W. Solenoid actuators were placed on two pieces of foam that were laid directly in front of the participants. The duration of each tap was set to 50 ms, and the interval between two taps was 300 ms. The participants responded by pressing foot switches (Figs 5 and 6).

### Experimental Design and Procedure

#### Unisensory tactile modality (Experiments 1–4)

A 2 (motion direction: leftwards or rightwards) × 2 (attention: focused or divided) factorial design was adopted. For relatively certain stimulus configurations, the two consecutively presented taps occurred on one hand: the first tap occurred at either the left middle finger or right middle finger, and the second tap occurred at the left forefinger/ring finger or right forefinger/ring finger. For more unpredictable stimuli, consecutive taps were randomly presented at any of the eight fingers, excluding the thumbs, considering that the thumbs have a larger receptive field and cause relatively higher variance during tactile perception^46–49^. The first two taps occurred simultaneously at the left ring finger/right middle finger or left middle finger/right ring finger. After 300 ms, the second tap was selected randomly and unpredictably from among the remaining fingers (i.e., excluding the thumbs and left ring finger/right middle finger or left middle finger/right ring finger, wherever the two simultaneous taps had occurred).

A typical trial proceeded as follows: A central cross (‘+’) appeared at the center of the monitor screen, followed by a blank interval of 300–350 ms that indicated the onset of the taps. When the taps had been presented, participants were required to respond within the time window of 1400 ms at the absolute position of the first tap of the tactile pair or with the direction of the tactile apparent motion for the given tactile pair. After the response, a gray ‘O’ symbol was shown to indicate a successful response (with no distinction for correct or incorrect responses). If no response was issued during the 1400 ms time window, ‘No response’ was shown on the screen. After every 60 trials, a break occurred, and the inter-trial-interval (ITI) was fixed at 2 s. For the ‘motion direction’ task, participants were required to discriminate the direction (leftwards or rightwards) of the tactile motion stream, in which the ‘congruent’ condition refers to the motion direction being congruent with the hand location (left hand vs. right hand). For the ‘localization’ task, participants reported the (relative) starting location of the first tap in the motion stream, and this location was relative to the location of the second tap in the motion stream. The ‘congruent’ condition in the localization task refers to the congruency between the hand location (left hand vs. right hand) and the relative location of the staring location (left vs. right) of the initial tap of the motion stream.

The number of trails was 240, and all the experimental conditions were randomized and counterbalanced for presentation. The participants were given short rests between each block of 60 trials.

#### Auditory-tactile modalities (Experiment 5)

**Figure 7:**
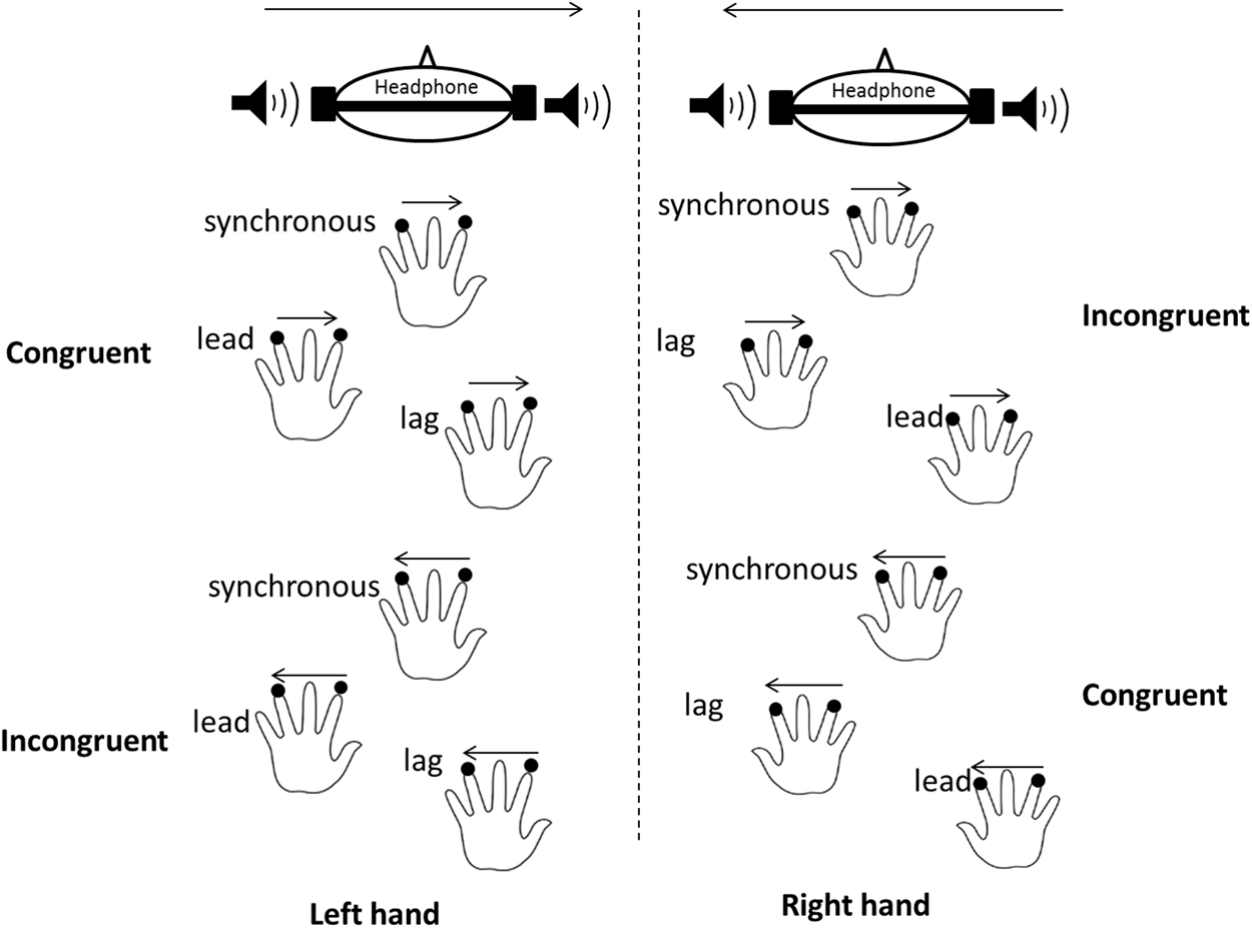
Schema for discriminating the direction of tactile motion with auditory apparent motion. Two taps were consecutively presented to the forefinger/ring finger and ring/forefinger of either the left or right hand. The auditory motion stream, which was composed of two beeps delivered to a headset with an SOA of 300 ms, preceded the onset of the first tap by 300 ms, was synchronous with the first tap or lagged the first tap by 300 ms. The arrows indicate the directions of tactile and auditory motion. ‘Congruent’ and ‘Incongruent’ refer to the directions of tactile and auditory motion being either the same or opposite.

The same touchpads were used as in Experiments 1–4. Here, we used a headset to deliver sounds. A 2 (left vs. right hand) × 2 (leftward vs. rightward motion) × 4 (conditions: baseline, auditory leading, auditory synchronous and auditory lagging) factorial design was adopted. In the baseline condition, only tactile motion stream appeared on either hand without concurrent beeps. The direction of auditory motion was either congruent or incongruent with the direction of tactile motion (presented to the left or right hand from the ring finger/forefinger to forefinger/ring finger). The duration of each tap was 50 ms, and the interval between two taps was 300 ms. For auditory leading or auditory lagging conditions, the onset of the first beep was leading or lagging the onset of the first tactile stimulus by 300 ms. The SOA between two beeps (60 dB, 50 ms, 1000 Hz) was 300 ms. The remaining timing parameters were the same as in Experiments 1–4. There were 480 trials in total. Participants took a break every six blocks (each block having 80 trials). Participants were required to discriminate the tactile motion, irrespective of the beeps (Fig. 7).
